# Patient Accessible Electronic Health Records: Exploring Recommendations for Successful Implementation Strategies

**DOI:** 10.2196/jmir.1061

**Published:** 2008-10-31

**Authors:** David Wiljer, Sara Urowitz, Emma Apatu, Claudette DeLenardo, Gunther Eysenbach, Tamara Harth, Howard Pai, Kevin J Leonard, Canadian Committee for Patient Accessible Health Records (CCPAEHR)

**Affiliations:** ^11^BC Cancer Agency—Vancouver Island CentreVictoriaBCCanada; ^10^School of Health Information ScienceUniversity of VictoriaVictoriaBCCanada; ^9^Department of SurgeryFaculty of MedicineUniversity of British ColumbiaVancouverBCCanada; ^8^Odette Cancer CentreSunnybrook Health Sciences CentreTorontoONCanada; ^7^Centre for Global eHealth InnovationUniversity Health NetworkTorontoONCanada; ^6^Department of Health PolicyManagement and EvaluationFaculty of MedicineUniversity of TorontoTorontoONCanada; ^5^E-Health TechnologiesGrand River HospitalKitchenerONCanada; ^4^Rollins School of Public HealthEmory UniversityAtlantaGAUSA; ^3^Oncology Education ProgramPrincess Margaret HospitalUniversity Health NetworkTorontoONCanada; ^2^Department of Radiation OncologyUniversity of TorontoTorontoONCanada; ^1^Knowledge Management and InnovationOncology Education/Radiation Medicine ProgramPrincess Margaret HospitalUniversity Health NetworkTorontoONCanada

**Keywords:** Electronic Health Record (EHR), Personal Health Record (PHR), medical records, recommendations, health planning guidelines, access, access to information

## Abstract

**Background:**

Providing patients with access to their electronic health records offers great promise to improve patient health and satisfaction with their care, as well to improve professional and organizational approaches to health care. Although many benefits have been identified, there are many questions about best practices for the implementation of patient accessible Electronic Health Records (EHRs).

**Objectives:**

To develop recommendations to assist health care organizations in providing patients with access to EHRs in a meaningful, responsible, and responsive manner.

**Methods:**

A Patient Accessible Electronic Health Record (PAEHR) Workshop was held with nationally and internationally renowned experts to explore issues related to providing patient access to the EHR and managing institutional change.

**Results:**

The PAEHR Workshop was attended by 45 participants who discussed recommendations for the implementation of patient accessible EHRs. Recommendations were discussed under four subject domains: (1) providing patient access to the EHR, (2) maintaining privacy and confidentiality related to the PAEHR, (3) patient education and navigation of the PAEHR, and (4) strategies for managing institutional change. The discussion focused on the need for national infrastructure, clear definitions for privacy, security and confidentiality, flexible, interoperable solutions, and patient and professional education. In addition, there was a strong call for research into all domains of patient accessible EHRs to ensure the adoption of evidence-based practices.

**Conclusions:**

Patient access to personal health information is a fundamental issue for patient engagement and empowerment. Health care professionals and organizations should consider the potential benefits and risks of patient access when developing EHR strategies. Flexible, standardized, and interoperable solutions must be integrated with outcomes-based research to activate effectively patients as partners in their health care.

## Introduction

There has been a growing interest in, and demand for, harnessing the power of electronic health records (EHRs) beyond just the delivery of care. The demand has arisen in part from the trend toward consumerism in health care. Patients and the public are no longer satisfied with the status quo and a growing wave of public and patient expectation is mounting [1-6]. Health care organizations are also realizing the need for patient accessible health records (PAEHRs) on a number of different levels including improving the patient experience, supporting patients with chronic conditions, improving transparency, increasing referral rates, and ensuring the continuity of care beyond the hospital walls. In addition, there is the growing global trend of adopting legislation to ensure that patients are able to access, review, and amend their medical record [7-18]. The coupling of these social and professional trends with new technologies that provide ubiquitous access to health information offers tremendous opportunity to transform the delivery of care.

A white paper from the American Association of Medical Informatics outlined the potential barriers to and benefits for the adoption of personal health records (PHRs) not only for patients, but also for health care organizations. There are a number of barriers to overcome, including privacy and security issues, change management issues, and the lack of basic infrastructure such as EHRs [19]. At the same time, potential benefits for patients include better access to health information, increased ability to self-manage chronic health conditions, increased medication tracking and safer prescription renewals, etc., and improved connections for patients and providers [19,20]. Potential benefits for organizations and health professionals include increased patient satisfaction, continuity of care, and improved standardization of care as organizations streamline processes and information to address this change in clinical practice [19,20].

In recognition of the potential benefits, many strategies and approaches have been developed to record electronically health and medical information and allow for electronic access to this information, most commonly through the Internet [21-25] and portable solutions such as CD-ROMs, mobile phones, and USB devices [26]. In addition, several pilot studies have demonstrated that many patients would review and interact with their medical record on an ongoing basis if the record was made available to them [2,5,6,16,27]. Many researchers and health care organizations have begun to implement pilot projects to test the concept of PAEHRs, but, with some notable exceptions [2], very few have been able to overcome all of the operational barriers to integration with clinical practice [21]. A few organizations, such as the Markle Foundation, have begun to establish basic principles for patient access to the EHRs [28,29], but there are very few standards, guidelines, and roadmaps for both the IT and clinical adoption of PAEHRs [20].

Mechanisms, in the form of policies and procedures, are therefore necessary to ensure success in moving towards a system that supports wide-scale use of PAEHRs. In an attempt to meet this need, the Canadian Committee for Patient Accessible Electronic Health Records (CCPAEHR) undertook a two-part project with the intent of (1) scanning the country to determine hospital readiness for the implementation and use of PAEHRs [30] and (2) assembling a PAEHR Workshop of key stakeholders in the field of the EHR and PAEHRs. The CCPAEHR is a group of Canadian researchers, clinicians, information specialists, and educators working together to promote patient access to and involvement with electronic health records. This paper presents the findings of the PAEHR Workshop. The results from the national survey have been published separately [31].

## Methods

### PAEHR Workshop

In response to the need for recommendations around the implementation of patient accessible electronic health records, a PAEHR Workshop was held in Toronto, Canada in October 2006 with nationally and internationally renowned experts. This workshop was designed to explore issues related to providing patient access to the EHR and managing the requisite institutional change. The objective was to develop draft recommendations that would assist health care organizations in providing patients with access to EHRs in a meaningful, responsible, and responsive manner.

The PAEHR Workshop involved the following steps: (1) a working group from an expert body, the CCPAEHRs, was established; (2) a national survey was conducted; (3) based on the published literature and the survey [2,5,32], a framework for patient access was developed; (4) four subject domains were identified; (5) subject briefs were created by experts in the working group; (6) the briefs were then provided to the entire CCPAEHR group for content validation; (7) national and international experts were identified through literature reviews and professional networks and invited to participate; (8) invited experts were sent copies of the subject briefs for their review and input; (9) the briefs were circulated prior to the workshop and participants were asked to review the materials and identify their area(s) of expertise; (10) presentations were delivered by experts in each of the domains and then participants broke out into working groups co-facilitated by the invited experts and CCPAEHR members to develop recommendations in their domain (proceedings were recorded by two transcribers per session); (11) the recommendations were presented to the larger group and discussed; (12) the CCPAEHR working group then analysed and summarized the discussion and drafted the initial report; (13) the report was sent to all participants for content validation; (14) two members of the research team then analyzed the recommendations and workshop notes for emergent themes.

### Terms and Definitions

For the purposes of the workshop, the definition of terms was intended to be as broad and as inclusive as possible, while maintaining the focus on patients accessing EHRs. EHR was defined as “a computerized record of a person’s health and/or medical history. This record may contain a person’s full health and medical record, or can be used for certain records, such as lab results, in conjunction with a more traditional paper-based patient chart” [19].

The concept of a PAEHR partly overlaps with the concept of a Personal Health Record (PHR), although there are some important differences. While there is no universally accepted definition of a PHR, it is important to delineate where the concepts overlap and where they diverge. The definition of PHR itself is controversial. In some concepts, the PHR includes the patient’s interface to a health care provider’s EHR while, in others, PHRs are thought of as being any consumer/patient-managed health record. A report from the National Committee on Vital and Health Statistics has noted, “This lack of consensus makes collaboration, coordination and policymaking difficult. It is quite possible now for people to talk about PHRs without realizing that their respective notions of them may be quite different” [33].

The *Connecting for Health* Personal Health Working Group sponsored by the Markle Foundation defines PHRs as follows:

The Personal Health Record (PHR) is an Internet-based set of tools that allows people to access and coordinate their lifelong health information and make appropriate parts of it available to those who need it. PHRs offer an integrated and comprehensive view of health information, including information people generate themselves such as symptoms and medication use, information from doctors such as diagnoses and test results, and information from their pharmacies and insurance companies [29].

The definition promoted by the American Health Information Management Association (AHIMA) is similar, but it stresses that the PHR is not simply a patient view on EHR data:

The personal health record (PHR) is an electronic, universally available, lifelong resource of health information needed by individuals to make health decisions. Individuals own and manage the information in the PHR, which comes from the health care provider and the individual. The PHR is maintained in a secure and private environment, with the individual determining the rights of access. The PHR is separate from and does not replace the legal record of the provider [34].

Tang and colleagues defined personal health records more broadly:

An electronic application through which individuals can access, manage, and share their health information and that of others for whom they are authorized, in a private, secure and confidential environment [19].

In the same article, the authors distinguished a “tethered” PHR (bound to a certain organization) from a “stand-alone” PHR and the ideal “interconnected” PHR.

PHRs, according to many definitions, do not have to be linked or integrated, either directly or indirectly, with clinical systems such as EHRs (they can be “stand-alone” PHRs), which is where the concept of PAEHR differs from the PHR concept.

For the purposes of the PAEHR Workshop, the definition of PAEHRs was narrowed to focus on patient access to provider-held, electronic records (in full or in part), regardless of the type of application that is used to provide access. As such, PAEHRs partly overlap with some PHR definitions (particularly “tethered” PHRs as defined by Tang and colleagues [19]).

### Workshop Participants

The issues related to PAEHRs traverse a number of areas of expertise. For this reason, a small group of experts in PAEHRs, as well as experts in other domains such as clinical practice, privacy, health care administration and policy, research, eHealth, information technology, consumerism, and patient advocacy were invited to participate in the workshop. Participants were sought from as many provinces as possible, and several international participants were invited in order to have heterogeneous viewpoints on a wide range of issues. To facilitate the process of inviting international experts, the workshop was held as a satellite event to a major international conference on Internet in medicine (Mednet 2006). Experts were identified through published literature, as well as nominated through the CCPAEHR committee. Experts were selected by reviewing their experience in various domains, their knowledge of the subject domains, their participation in related national and international initiatives, and their publication records (Table 1). Despite the attempt to have geographic, academic, and clinical diversity amongst workshop participants, many of the identified experts came from a few regions and organizations in the country where active work in the field was being undertaken. In addition, many of the clinical experts identified were working in the area of oncology. Attempts were made to broaden representation from medical disciplines and international experts were identified and invited to ensure a broader representation from across disciplines and specialties.

**Table 1:** PAEHR Workshop participants inclusion/exclusion criteria

Inclusion/Exclusion Criteria	
1.	Identified as a domain expert by a Member of the Canadian Committee for Patient Accessible Health Records (CCPAEHR).
2.	Researcher actively addressing issues related to patient accessible EHRs.
3.	Participant in a clinical implementation of patient accessible EHRs.
4.	Experts working in closely related issues such as patient education and privacy.
5.	Researchers addressing patient empowerment or patient advocacy issues.
6.	Clinical staff with an active interest in patient access to their health information

Members of the lay public did not participate in the PAEHR Workshop, because the workshop was intended as a first step in the identification of issues and potential recommendations. Obtaining patient involvement and public engagement was determined to be part of subsequent phases of this ongoing initiative.

### Four Subject Domains

The working group identified four major subject domains for PAEHRs through literature reviews and discussions with the CCPAEHR committee: (1) providing patient access to the EHR, (2) maintaining privacy and confidentiality related to the PAEHR, (3) patient education and navigation of the PAEHR, and (4) strategies for managing institutional change. For each subject domain, a briefing note was created based on the issues articulated in Leonard’s *A Prescription for Patience: A Guide to Improving Our Healthcare System* [35]. Each briefing note contained a general summary, a list of topics of interest, a reference list, as well as draft recommendations for each subject domain (Multimedia Appendix 1: Briefing Documents).

## Results

The PAEHR Workshop was attended by 45 participants and renowned experts from the United States, Canada, Spain, Iceland, and the Netherlands. Participants contributed to the development of recommendations through moderated breakout and discussion sessions. The discussions for each subject domain, summarized by the research team and validated by the participants themselves, were as follows:

### Patient Access to the EHR

Most participants agreed that access to the EHR is a fundamental patient right and that the implementation of PAEHRs should not be delayed. However, there was little agreement on exactly how access should be provided. There were two general but opposing approaches which emerged. The first was to provide access to only the “relevant” content in the EHR. Ideally, this clinical information should be coupled with tailored educational materials to help people meet their information needs. However, there were some participants who thought patient access to these results should only be provided after being vetted by a physician, or viewed only in the presence of a health care professional, as an approach to managing the anxiety that may transpire from accessing results perceived as “bad news”. The second approach was to provide open access to all information contained within the EHR and allow the patient or their proxy to decipher what information they feel to be relevant. In this approach, educational information could also be linked to a fully accessible PAEHR; however, the tailoring of information with a broad release of results was perceived to be an enormous barrier to adoption of this approach.

With reference to the EHR, there was general agreement that access should include the ability to make entries into the EHR. Patients should be both receivers and contributors of information. Allowing for this type of patient annotation could result in a feeling of “ownership” on the part of the patient. However, there were concerns about who would be responsible for reviewing or monitoring patient entered data and the potential for professional liability if patient entered data was not addressed in a timely manner.

### Privacy and Confidentiality

There was agreement among participants regarding the necessity to adopt and support one standard with respect to ownership and/or custodianship of the EHR and its content. Traditionally, the patient record has existed under the control of the provider or treating institution/organization. As patient access continues to increase, this would ultimately result in a culture shift related to the control of health information. Mechanisms need to be in place to help manage the potential conflicts resulting from territorialism and protect providers of health information from the risks of sharing ownership of information with their clients. In the short run, providers may be reluctant to give up what has traditionally existed in their domain.

Regular and ongoing access by patients to the EHR demands the development of policies and procedures related to record management. Patient records should be audited regularly to ensure the accuracy, integrity, and quality in the record, especially in situations in which patient entries are permitted and incorporated into the record. Furthermore, policies need to be in place regarding the retention of information. The emergence of patient portals and the ability to customize patient views may result in a unique set of challenges. In addition, clear statements of where the institutionally-based EHR ends, and the patient portal begins will need to be articulated.

### Patient Education & Navigation

With respect to EHRs and PAEHRs, it was agreed that patient education can be understood as either a means of educating patients on how better to understand and use EHR data, or it can be understood as the information necessary to educate people on what the EHR is, what it contains, individual rights regarding the EHR, and the potential benefits of accessing the EHR. It was agreed that the provision of education within the EHR should not be used in lieu of information provided by health care providers, but rather as a supplemental source.

When providing access to the EHR, the provision of educational support should be available to all, but presented so that individuals who do not need the resources are not inundated by them. This could be accomplished by embedding links to credible educational sites. It was agreed that the standardized educational materials in relation to elements of the EHR should be adopted.

### Institutional Strategies for Change

In order to succeed in wide-scale adoption and implementation of PAEHRs, systems need to be in place to help health care providers, primarily clinicians, feel less threatened by the introduction of this new technology. The benefit of the innovation needs to be demonstrated through research and the development of evidence-based protocols.

Accepting the cost of change was highlighted as another step towards successful change management. Unless institutions are willing to cover the financial costs associated with the adoption of these new technologies, it is unlikely that they will succeed. There needs to be the acknowledgement that workload may increase in the short run. There needs to be continuous organizational reassurance that the increased burden will not continue in the long run and that support will be provided.

The success of institutional change is also dependent on the specific drivers for change. The importance of a physician champion clearly emerged. However, a culture shift is required recognizing that access to medical records is a fundamental right of every patient. In an institution committed to patient-centered care, making patients the drivers of change may help to guarantee success. Unlike clients in other industries, patients have traditionally experienced a power imbalance in health care. Now that patients are becoming more empowered, health care systems need to develop means of meeting the consumers’ demands and needs—providing PAEHRs would be an important first step.

### Recommendations for PAEHR Implementation

From the discussions and briefing notes, each workshop group developed a set of draft recommendations. These recommendations were presented to the group for discussion and approval and then they were reviewed a second time once the final report was completed. The recommendations outline priority areas for each of the subject domains (Multimedia Appendix 2: Subject Domain Recommendations). Although the recommendations were developed for each of the subject domains, there was, in fact, a great deal of overlap, and several important themes that transcend the domains were identified by the research team:

1. *National Infrastructure*: There is a need for national standards and guidelines that will ensure that patient-centered care is delivered nationally. The infrastructure will include not only the required IT networks, but also the infrastructure to support the development and dissemination of policies, procedures, security protocols, and educational standards. In addition, the infrastructure should engage the public, raise awareness, and promote knowledge sharing and patient advocacy.
2. *Security and Confidentiality*: Security and confidentiality must be protected according to national standards, but at the same time, a paradigm shift is required so that health care organizations create a culture of custodianship, rather than ownership, of patient data. This shift will be achieved by creating models of shared control between health care professionals, patients, and the public. Health care organizations need to be confident they can manage the additional risk exposure in sharing electronic patient information with their users. Patients should have the ability to control the flow of their clinical data and to delegate access to the data.
3. *Flexible, Interoperable Solutions*: No one solution will fit all of the diverse health care settings; therefore, flexibility is required at all levels of the implementation of PAEHRs, including: (1) flexibility for diverse clinical practices; (2) flexibility for diverse organizational cultures and approaches to clinical care; (3) flexibility for diverse patient groups; (4) flexibility to accommodate patient choice and promote a patient-centered model of care; and (5) interoperable solutions to ensure the continuous flow of personal health information.
4. *Education*: Education is required at all levels. Education materials should be developed to support clinicians through the paradigm shifts and cultural changes that are required for patient-centered care models. Public education is required to raise awareness of fundamental rights to access health data. Patient education is also required to help patients understand the nature of the health record itself, including methods of reporting results and tests, and, at the same time, education is required to help patients understand what their clinical data means to them and how they can manage their care to ensure the best possible health outcomes. Health care administrators need to be educated on how to deliver and manage PAEHR systems and the costs associated with such practice.
5. *Research and Evidence-Based Practice*: Little is known about the potential risks and benefits of PAEHRs. Research should be a fundamental component of implementing PAEHRs and should focus not only on evaluation research to ensure that the best possible systems are put in place, but also on outcomes research to measure the health benefits in order to identify the real risks and the true benefits.

## Discussion

Participants of the PAEHR Workshop did support the concept of patient access to electronic health records; however, many important issues and concerns were expressed. The themes emerging from this PAEHR Workshop were, on a high level, similar to themes articulated for PHRs in general: the focus on the need for interoperable solutions for information exchange to avoid building “information islands” [19], the need for education at all levels [19], the need for research, and the need to build systems that respond to audiences with diverse needs to eliminate barriers to patient use [36]. However, within the specific context of patient access to the EHR, there were some important shifts in focus and concerns about national infrastructure and security, privacy, and confidentiality that emerged. This PAEHR Workshop also made a substantial contribution in creating a draft framework consisting of 22 specific and practical recommendations. Although there is a great deal of work to do in terms of validating the recommendations from many different perspectives, including that of the patient and the public, this workshop represented an important step towards the widespread implementation of PAEHRs.

It was clear from the PAEHR Workshop that there are many issues surrounding PAEHRs for which there was still little agreement or great uncertainty. There was a lack of agreement around fundamental issues such as how much of the EHR should be provided. Many participants thought that patients do not need access to certain results, despite the fact that several studies have illustrated that patients would like full access to all elements of their EHR [6]. In the Canadian context, the Supreme Court ruling in 1992 on McInerney vs MacDonald states that, while not an absolute right, patients have a right to access their personal health information in all but a few circumstances based on the fiduciary relationship of the patient and doctor [37]. In other countries such as the UK, the argument of patient access based on the fiduciary relationship has not been upheld, but laws have been put in place to ensure that patients have appropriate access [37]. Even within Canada, there are very few standards in the practice of providing access to personal health information [31]. The McInerney vs MacDonald ruling came before the widespread use of EHRs, and therefore the courts have not clarified many issues that have become pertinent because of the use of new technologies. The discussion of the PAEHR Workshop reflected the complexity of the issues and the diverse approaches and attitudes toward providing patients with access to their own personal health information.

There was also an important discussion and debate about when results should be provided—in real-time, after physician approval, or after a specified time delay. A balance must be struck between making the information available to patients in a timely fashion that supports self-managed care and patient safety so that patients are not unduly stressed by complex and ambiguous information. However, it is evident that the health care community is currently divided on this issue.

It is clear from the recommendations that emerged from this workshop that flexible solutions will be required to meet diverse organizational structures and patient populations. In addition, research that extends beyond the evaluation of delivery systems is desperately needed to provide some cornerstones that will support future developments. Research is required in every domain of PAEHRs. Although a great deal of work has been completed in testing the efficacy of the idea and the usability of certain applications [2,16,24,38], little research has been completed that demonstrates the benefits or potential risks of EHRs.

Infrastructure is required if PAEHRs are going to be successfully implemented into the health care system. Although PHRs can be a combination of data that is both entered by patients and pulled from existing clinical systems, PAEHRs must be incorporated in the clinical roadmaps that include the development of EHRs. The IT infrastructure, however, is only one barrier to adoption. The need for policies, procedures, and clinical infrastructure that support PAEHRs is evident. In addition, changes in clinical practice may be required to reap the full potential from PAEHRs. As has been pointed out, implementing a “disruptive” technology will take time [20], and technology adoption models clearly predict increases in resource utilization before the benefits of new technologies are realized [35].

Finally, it is clear from this workshop that a national—perhaps embedded into an international—debate is required regarding the relative risks and the potential benefits of PAEHRs. The introduction of PAEHRs will require the allocation of resources and major changes in clinical practice. At the same time, there are potential risks that are not yet well understood. There are privacy, confidentiality, and security issues that must be managed. Levels of security, for example, could become so tight that PAEHRs could become virtually unusable, and thus the ratio of acceptable risk versus potential benefits must be established. Public education is required, and awareness of patients’ rights and responsibilities in changing health care models must be raised. The public and consumer demand for PAEHRs will be a major determinant of how clinicians and health care organizations respond [4], and without patient advocacy and clinician champions, the numerous barriers to adoption may continue to stand in the way of widespread adoption.

There were a number of key limitations in the design of the workshop. Although many of the findings relate to key principles, the discussion focused primarily on the realities of the Canadian health care system and, in particular, to a few organizations focused on developing patient accessible electronic health records. This was not unexpected as a recent Canadian survey indicated that very few organizations were ready to provide online access to the EHR [31]. Furthermore, while patient advocacy viewpoints were expressed, patient viewpoints were not well represented as part of this consensus building process. Thus the opinions emerging from this workshop represent those primarily from representatives of the health care sector and academic fields. Since the development of patient accessible EHRs is still quite new in this context, developing recommendations was an ambitious goal for the PAEHR Workshop. The development of a framework and preliminary recommendations, while an important step forward, still need to be tested within multiple practice settings and validated through a public and patient engagement process.

### Conclusions

Patient access to EHRs is a fundamental patient right, and health care professionals and organizations must move in a responsive and responsible manner to provide this access. There are many issues that need to be addressed, and in the absence of research and generalizeable evidence, organizations are faced will a cadre of difficult and complex operational issues. Targeted research is essential, and at the same time, coordinated, national efforts are required to provide the necessary infrastructure for PAEHRs. Flexible, standardized, and interoperable solutions are essential for ensuring that PAEHRs support integrated, comprehensive care. Providing access to EHRs is a vital next step in activating patients in their care and improving the health system on a profound scale. The challenge remains for organizations, policy makers, clinicians, and patients to respond to this need and put these recommendations into practice.

## Multimedia Appendix 1

Briefing documents

## Multimedia Appendix 2

Subject domain recommendations

## Abbreviations

AHIMA: American Health Information Management Association
CCPAEHR: Canadian Committee for Patient Accessible Electronic Health Records
CHI: Canadian Health Infoway
CIHR: Canadian Institute for Health Research
EHR: electronic health record
PAEHR: patient accessible electronic health record
PHR: personal health record
