# Supplementary material for: Patient Accessible Electronic Health Records: Exploring Recommendations for Successful Implementation Strategies
Source: J Med Internet Res. 2008 Oct 31;10(4):e34. doi: 10.2196/jmir.1061 (PMC2629367; doi:10.2196/jmir.1061)
Supplement: Supplementary file 1 [file jmir_v10i4e34_app1.pdf]

Multimedia Appendix 1: Briefing Documents

Textbox 1:

Policy Brief

Patient Access to the Electronic Health Record

Speaker: James Walker
Co-Facilitator: Howard Pai

Introduction:

The utilization of information technology to facilitate patients' access to their health records can have several potential benefits to patients, health care providers and the healthcare system. Examples of benefits to patients may include more convenient and timely access to health records, the ability to schedule appointments and maintain an electronic diary of their health history. The provision of electronic health records to patients may also facilitate communication between patients and health care providers and engage patients more in their own healthcare. Ultimately, we envision that providing patient access to their electronic health records will be a determinant of health outcome and satisfaction with care.

One necessary condition for bridging technology and patient access is a well-thought out strategy and implementation. Investment in technology cannot take place without first understanding needs, barriers, gaps, process issues, the health delivery system and the information requirements for the future.

Topics of interest and research questions:

- Peer-reviewed evidence.
    - What does the peer-reviewed evidence demonstrate? What are the benefits and potential harms of patient access to electronic health records?
- Needs assessment.
    - Is there a need for this technology? If so, who will benefit and how?
- Buy-in.
    - Who are the major stakeholders and are they involved? Who will lead this initiative?
- Patient empowerment – Will the patient armed with access to their own health records, coupled with credible health information tailored to their needs, become a more active partner in the health care encounter?
    - What will this mean for treatment decision-making?
    - Is the health care system ready to support empowered patients?
    - What mechanisms need to be in place to provide and support patients access to their health records?
- An empowered patient will necessarily result in changes to the patient-physician relationship.
    - What type of professional support is necessary to support physicians and other healthcare providers in communicating with the "new empowered patient"?
- Information technology will enable patients in remote areas to more easily integrate into larger health care systems.
    - Does access to one's own health care information facilitate communication with experts that may not be local?
    - Can Telehealth support having the patient at the centre of the care team in either the role of decision maker and/or gatekeeper of information?
- Providing patients with access to their health record may result in improved information sharing capabilities among different levels of healthcare personnel and patients.
    - What mechanisms need to be in place to ensure that the patient shares the appropriate and accurate information with each health care provider?
    - Who should have access to the EHR? And how will this be regulated?
    - Should access be provided to the full record, or only relevant sections of the EHR?
- Convincing consumers and providers to change old habits and to adopt using information technologies to access health care information.
    - How and when do we make patient accessible records the standard of care?
    - Who is going to pay for it: consumers, government, health care providers, and/or private industry partnership?

Supporting Papers:

Cimino, J. J., V. L. Patel, et al. (2001). "What do patients do with access to their medical records?" Medinfo 10(Pt 2): 1440-4.

Anonymous (2004). "Patients accessing Web-based medical records." Internet Healthcare Strategies 6(6): 1-4.

Pyper, C., J. Amery, et al. (2004). "Patients' experiences when accessing their on-line electronic patient records in primary care." British Journal of General Practice 54(498): 38-43.

Masys, D., D. Baker, et al. (2002). "Giving patients access to their medical records via the internet

Policy Brief
Maintaining Privacy and Confidentiality

Speaker: Miyo Yamashita
Co-Facilitator: Tiffany Jay

General Summary:

Many industries have implemented safe, non-compromising online platforms to relay and exchange consumer information with the support of their clients. These efforts have created a framework in which to consider security when moving forward with patient access to the electronic health record (EHR). However, reflecting the high sensitivity of personal health information, privacy and security remains critical consideration in moving towards this goal. Balancing the security concerns is the movement by legislators to provide patients a statutory right to access their personal health information.

Since the implementation of the HIPAA Privacy Rule in the US, access complaints represented the third most common type received by the oversight body. Many of these complaints related to high cost for access and rigid restrictions on access time and place imposed by providers. Electronic access may provide an avenue to alleviate these concerns. However the other volume of concerns relate to denial by providers of access to all or parts of the patient's record. In addition, providers have reported a reluctance to include sensitive or controversial information in their documentation for fear that patients may complain or demand that information be changed.

Several provinces across Canada have similarly accorded a statutory right for patients to see their health information. These statues provide limited opportunities for the health provider to refuse the patient's right of access, for example where it is believed that access may cause harm to the patient or another individual. Providers are typically required to make reasonable efforts to make the information accessed understandable to the patient (i.e. provide explanation of acronyms or medical terminology).

Other rights and obligations with implications for electronic patient access include records retention mandates, the need to put into place appropriate safeguards (including security) and requirements for information to be accurate and complete permitting the patient to request correction when this standard is not met.

Topics of interest:

- Who should develop operational privacy standards for patient access? Institution? Province? Federal?
- Risk of harm – Is there information to which patient shouldn't have immediate access?

- Access Provisioning - Should patients be permitted to delegate access to their record (friends, family)?
- Accuracy & Requests for correction – How do institutions manage concerns brought forward by patients? What effect will patient access have on the record-keeping behaviour of care providers?
- Usability – What is the institutions responsibility for making the information presented meaningful and understandable?
- Storage & Retention – Where should information be retained? For how long? How does patient-generated information relate to the medical record including legal and regulatory standards?
- Safeguards – What security systems need to be in place before an institution can provide patients electronic access to the records? What's the appropriate balance between the obligations of the hospital and the autonomy of the patient?
- Limiting Use & Disclosure – Should limits be considered around how information collected in the course of providing patient access will be used (e.g. research use of identifiable utilization data or journal entries)?
- Challenging Compliance - What recommendations need to be in place for situations in which confidentiality is breached?

Supporting Papers:

Brown, Bob (2006) "The Patient Access and Request for Amendments Provisions of the HIPAA Privacy Rule." Journal of Health Care Compliance **8**(3): 37-79.

Hassol, A., Walker, J., Kidder, D., et al. (2004) "Patient Experience and Attitudes about Access to a Patient Electronic Health Care Record and Linked Web Messaging." Journal of the American Medical Informatics Association **11**(6): 505-513.

Kimber, Tim (2006) "HOW TO… Manage Access to Patient Notes" General Practitioner 6/9/2006: 45.

Masys, D., Baker, D., Butros, A. Cowles, K. (2002) "Giving Patient Access to Their Medical Records via the Internet: The PCASSO Experience." Journal of the American Medical Informatics Association **9**(2): 181-191.

Tjoral, A., Tran, T. & Faxvaal, A. (2005). "Privacy vs. Usability: A Qualitative Exploration of Patients' Experiences With Secure Internet Communication With Their General Practitioner." Journal of Medical Internet Research **7**(2): e5.

Policy Recommendations:

- At minimum, each institution wanting to adopt the use of an EHR must adhere to all provincial standards, or where the portal crosses provincial boundaries, to the highest relevant jurisdictional standard.

- Procedures should be in place to respond to questions and requests for correction about the accuracy of the record and support care providers in responding to challenges regarding accuracy.
- Security safeguards must be given priority in balance with the need to provide a usable system.
- In determining what information is accessible, institutions must engage in clinical consultation to ensure that the risk of harm is mitigated when providing access.
    - This may include setting standards around appropriate delays before posting information to a patient portal to provide the provider an opportunity to review results.
- Education & support are key success component in providing patient access - information provided should be understandable and meaningful.

Policy Brief
Patient Education and Navigation of the Electronic Health Record

Speaker: Steve Ross
Co-Facilitator: Audrey Friedman

General Summary:

Providing patients with access to and involvement with the electronic health record (EHR) is, from a technical point of view, relatively straightforward. However, as the scope of the EHR expands from a clinical management tool to a patient resource and self-management tool the complexities and difficulties quickly arise. If the access is designed to engage and empower patients to participate in their care and better navigate the health system, then perhaps access to clinical data is not in fact sufficient. Further information and education is required to empower patients to participate in their care and better navigate the health system.

There are several potential benefits to be achieved through educating patients on and through the EHR. The benefits include increased patient engagement, satisfaction with their care and overall quality of life. It also provides a platform for web-based interventions to promote patient activation through education around issues of chronic disease management, including adherence to care plans, self-management and self-care strategies. In addition, it has the potential to increase patient safety through awareness and improve patient-provider communications.

From a clinical practice perspective, there are also a number of important benefits to be realized. Clinical management benefits may include a reduction in clinical visits, reduced phone calls to the clinic, reduced wait times, increased patient safety, and improved community integration and outreach. In addition, training and education will play a significant role in the development of patient information systems due to the fact that most patients do not have a sense of what comprises their health record let alone the value of the information contained therein.

Consequently, if we advocate that patients must be more pro-active in their health management and we propose that they access an information system containing their EHR, then we must ensure that patients receive the education they need to best navigate the EHR. Some research has been conducted to demonstrate the importance of targeting and tailoring health messages and the EHR has been identified as a powerful platform that could be harnessed for this purpose. However, there are many clinical, legal, security, educational and social issues which need to be resolved and further research is required to assess impact on clinical practice.

Topics of interest:

- Training and education for patients and healthcare providers.
  - What levels of education are required to ensure success adoption of accessible EHRs?
  - Should education be optional and sought out by users or mandated and enforced by professional organization, or prescribed by health care providers?
- Tailored vs. Targeted Information.
  - Is information tailored for an individual patient required?
  - How should information be tailored in the context of co-morbidities?
  - Should information be tailored for cultural and linguistic diversity?
  - What are the liabilities connected with tailored information?
- Maintaining content.
  - What systems need to be in place to ensure that educational content is up to date and accurate?
- Patient anxiety - having records available may cause patients to overreact.
  - What systems need to be in place to ensure that people understand the content of their record?
  - How to ensure that support mechanism are in place to support people who read potentially upsetting information in their record?
- What impact will access to records have on current workloads?
  - Additional studies are necessary to determine the impact on the timing of the provider-patient encounter.

Supporting Papers:

Kane, R. J. (2002). “Information is the key to patient empowerment.” Annals of Health Law **11**: 25-44.

Deber, R. B., N. Kraetschmer, et al. (1996). “What role do patients wish to play in treatment decision making?” Archives of Internal Medicine **156**(13): 1414-20.

Fowles, J. B., A. C. Kind, et al. (2004). “Patients’ interest in reading their medical record: relation with clinical and sociodemographic characteristics and patients’ approach to health care.” Archives of Internal Medicine **164**(7): 793-800.

Goldberg, H. I., J. D. Ralston, et al. (2003). “Using an Internet comanagement module to improve the quality of chronic disease care.” Joint Commission Journal on Quality & Safety **29**(9): 443-51.

Kreuter, M. and C. Skinner, *Tailoring: what's in a name?* Health Education Research, 2000. **15**(1): p. 1-4.

Kreuter, M. and C. Skinner, *Tailoring: what's in a name?* Health Education Research, 2000. **15**(1): p. 1-4.

Kreuter, M., et al., *Achieving cultural appropriateness in health promotion programs: targeted and tailored approaches.* Health Education & Behavior, 2003. **30**(2): p. 133-46.

Kreuter, M., V.J. Strecher, and B. Glassman, *One size does not fit all: the case for tailoring print materials.* Ann Behav Med, 1999. **21**(4): p. 276-83.

Policy Recommendations:

- Patients receiving access to their EHR should also receive access to educational material that will help them understand the elements of their record and any actions they might need to take. These actions may include adhering to care plans, self-management strategies, required follow ups, and additional information and support that could be accessed if required.
- Where possible information should be tailored for patient sub-groups and tailored for the individual clinical needs of patients. Tailoring should be based on clinical parameters and contextualized for patient issues.
- The information that is provided should reflect the needs of the target populations and should be culturally and linguistically relevant.
- The development of National Advisory Boards to develop standardized education content for use in the EHR. The standards should be linked with evidence-based guidelines developed by organizations. Development of standards (guidelines) on healthy living, disease management etc. to be used in conjunction with the EHR
- Funding mechanism in place to enable education necessary around access and understanding the EHR.
- Educational materials should also be targeted to family members and caregivers when appropriate.

Policy Brief
Institutional Strategies for Change

Speaker: Sam Marafioti
Co-Facilitator: Claudette DeLenardo

General Summary:

The implementation of an electronic health record system requires significant organizational change from constituents from all levels; consumers/patients, health care providers and administrators share a common vision. Change is hard. Change is about loss. It disrupts the normal functioning and practices of an organization. In order to support change in practice, the change must be perceived as an organizational priority. The ability promote change through an organization is dependent on decisions made by members of the system. People will adopt change if they believe that it will be a benefit to them. The implementation of an electronic health record poses one of the greatest changes to one's health care career. In order to ensure successful implementation and adoption, one must affect the attitudes of those individuals who are seen as "leaders" within systems and subsystems. The need to focus on people's willingness to accept change cannot be overstated.

Traditionally, health care providers view the health record as owned by an organization. With the implementation of PHIPA, this traditional way of thinking is being challenged. As a result, organizations must be responsive to changes that support patient access to their health information in a timely fashion. Patient access to the electronic health record to support engagement in self-care management adds another level of complexity to the health care system. Barriers to access include usability, navigation, health literacy and applicability.

Topics of interest:

- How do we bridge the communication gap between stakeholders (technology consultants, health care providers and consumers (patients)) involved in the development of technology applications for healthcare?
- What change management processes incorporate adoption and long-term sustainability of innovations?
- If patients are engaged in decision-making using technology applications how does it affect the patient-provider relationship? What is the result of this relational change on providers and their perceptions of "patient engagement"? How does this change affect roles within health care?
- How do we engage patients in care using the electronic health record in order to change the context of patient-provider relationship?
- How are patient-centric environments defined?
- Reduction in Canadian health care costs

Supporting Papers:

Clement, M. J. (1997). "The Barriers to Electronic Medical Record Systems and How to Overcome Them." Journal of the American Medical Informatics Association **4**: 213-221.

Hassol, A., Walker, J.M., Kidder, D., Rokita. K., Young, D., et al. (2004). "Patient experiences and attitudes about access to a patient electronic health care record and linked web messaging." Journal of the American Medical Informatics Association11(6), 505. 513

Mandel, J. L. e. a. (1998). "Electronic Patient-Physician Communication Problems and Promise." Annals of Internal Medicine **6**: 495.

Policy Recommendations:

- Need for research to develop applications that meet the navigation and usability needs of patients.
- Availability of fiscal and human resources for development, implementation and evaluation of change management processes
- Need for research to promote best practices for change management processes.
- Change practices are required to be embedded in the practices of the organization and supported by the management team, senior executive and board of directors.
