# Supplementary material for: Patient Accessible Electronic Health Records: Exploring Recommendations for Successful Implementation Strategies
Source: J Med Internet Res. 2008 Oct 31;10(4):e34. doi: 10.2196/jmir.1061 (PMC2629367; doi:10.2196/jmir.1061)
Supplement: Supplementary file 2 [file jmir_v10i4e34_app2.pdf]

Multimedia Appendix 2: Subject Domain Recommendations

Text Box 2: Subject Domain Recommendations

Subject Domain	Recommendations
Patient Access to the EHR	1. A framework needs to be defined whereby all Canadians should have access to at least some elements of their EHR in institutions where such a record exists if desired.<br>2. Within a pre-determined time period all Canadian health care facilities shall be responsible for providing high performance care.<br>2.1. The ability of providing high performance care is dependent on the presence of an EHR and patient participation.<br>2.2. Wide scale adoption of EHRs and PAEHRs will be achieved through the implementation of a business plan, rather than through "imposing" a need for them on institutions.<br>3. Providers and the public should be educated to increase awareness that patient charts (records) and the information contained therein belongs, at least in part, to the patient and that it can and should be accessed by them (similar to banking or credit card information).<br>4. The move towards empowering patients by providing them with access to their EHR/PHR, coupled with the necessary educational materials should become the basic standard of care.<br>5. Formal research is necessary in the field of PAEHRs in addition to implementing a change in current practice. The impact of PAEHRs should be studied in order to demonstrate true benefit and effect.
Privacy and Confidentiality	1. Implementation of the EHR requires a clear understanding and definition of custodianship/ownership of the EHR.<br>1.1. An explicit statement of who has custodial authority is required, and it is recommended that control be shared between the patient and the provider in a framework that is respectful of patients' privacy and autonomy.<br>2. There should be flexibility in terms of deciding which elements of the EHR should be accessible.<br>2.1. Patients should have a choice of what elements of the EHR they want to access.<br>2.2. Support should be provided to assist with understanding the content that patients want to access; at a minimum, this should include interpretation of test

	results.<br>3. Mechanisms need to be in place that ensure security and access control and/or management<br>3.1. Patients should have the ability to delegate access to the EHR<br>3.2. Assurances are necessary to ensure that content in the EHR is accurate and complete and patients are aware of institutional procedures to challenge the accuracy and completion of the information<br>3.3. A system of physical, technical and administrative protections or safeguards needs to be in place for both the patient and the institution. These standards should at a minimum meet current standards, but preferably exceed current standards<br>3.4. The organization should have standards for limiting patients' access to their information and a procedure for communicating these restrictions in compliance with relevant legal standards where such standards exist<br>4. Systems of data retention need to be clearly defined.<br>4.1. A formal system of how the data can be accessed and when it can be accessed needs to be in place.<br>4.2. For institutions that use both an EHR and a patient accessible portal/personal health record, there needs to be a clear understanding of where the official EHR ends and the portal/PHR starts. There may be different rules and requirements for which elements, and for how long, these need to be retained by the institution. This framework must be communicated to the patient.<br>4.3. For institutions that were using an EHR and have added a portal/PHR, current policies on access will need review and revision.
Patient Education & Navigation	1. Access to information in the EHR is more likely to result in better health if it is linked to credible educational and support materials and resources. These can include informational sources for individual learning, or a direct link to an educator or information specialists.<br>**2.** Providing patient access to the EHR creates unprecedented opportunities to capture patient experiences and to tailor educational materials to individual patient needs**.** Health care providers, and educators need to capitalize on these opportunities, which may result in improved health related and quality of life outcomes.<br>3. The information that is provided in EHR should reflect the needs of the target populations and should be culturally and linguistically relevant.

	4. Educational materials that are linked to the EHR should also be targeted to family members and caregivers where appropriate.<br>5. National standards should be developed to promote the dissemination of EHR related education materials to facilitate interoperability.<br>6. In addition to the educational materials that are necessary to understand the content of the EHR, a wide scale mechanism should be in place to enable the public education necessary for patients to access and understand the EHR.<br>7. Research is required to determine the most effective ways to engage patients in the care process in order to support behavioural change and improved health outcomes.

Institutional Strategies for Change	1. Institutions need to be proactive and accelerate the implementation and adoption of the PAEHR. Successful implementation is dependent of the following:<br>1.1. The creation of structured approaches to change management within organizations to support the development of a PAEHR. These approaches must meet the needs of the end users (including patients), and must be operationalized as a standard for development.<br>1.2. The development of change management activities to support patient access to the EHR, that include a comprehensive analysis of the cultural climate of the organization and identification of barriers that may prevent adoption of patient access to the EHR.<br>1.3. The development of mechanisms to help organizations manage/overcome barriers to implementation of the PAEHR.<br>1.4. The development of mechanisms that incorporate long-term sustainability and integration of PAEHRs into the clinical care process.<br>1.5. A national forum should be created for knowledge sharing with respect to managing institutional change in relation to the adoption of PAEHRs. This forum should be included as a component of an e-health summit or conference.<br>2. The successful adoption of new technologies is dependent upon embracing a holistic concept of patient-centred care.<br>3. The concept of "patient-centred care" needs to by operationalized and established as a central concept in the delivery of health services. The operationalization should include the right for patients to access their health information contained in their EHR.<br>4. Educational institutions that train health care providers should be encouraged to incorporate patient-centred concepts within their curricula.<br>5. Government<br>5.1. Government must expedite the e-health agenda as a priority in Canada and expend the required resources to support the development and implementation of interoperable EHRs.<br>6. Patient advocacy<br>6.1. Establish a grassroots patient movement to mobilize patient's awareness of the right to access their personalized health information to support self-managed care and decision-making. Establish a grassroots patient movement to mobilize patient's

	awareness of the right to access their personalized health information to support self-managed care and decision-making.<br>6.2. A national statement for patient awareness of rights to access the EHR needs to be developed and adopted as a standard within the delivery of health services.
